# Under-diagnosis of alcohol abuse: a descriptive study in a psychiatric hospital

**DOI:** 10.1192/j.eurpsy.2023.729

**Published:** 2023-07-19

**Authors:** A. Lopez Fariña, U. López Puentes, I. Alonso Salas, C. Gonzalez Navarro, A. Bilbao Idarraga, L. Morado Sansegundo, U. Ortega Pozas, C. Aran Cisneros, B. Samso Martinez, R. F. Lopez Brokate, T. Ruiz de Azua Aspizua, E. M. Garnica De Cos

**Affiliations:** Red de Salud Mental de Bizkaia, Zamudio, Spain

## Abstract

**Introduction:**

Incidence of alcohol abuse in our country is high, although it is still under-diagnosed and under-treated. The WHO estimates that a total of 3.3 million deaths worldwide per year are related to alcohol consumption.

**Objectives:**

The main objective is to describe the pattern of alcohol consumption in a sample of patients who are admitted to our psychiatric hospital for different reasons, relating with previous diagnoses.

**Methods:**

A retrospective observational descriptive study was carried out in the acute care unit of the psychiatric hospital, after approval of the corresponding protocol by the ethics committee. All patients admitted to this unit during a three-month period were taken as a sample. During admission, sociodemographic data, drug use, treatment type and time and previous diagnoses were collected.

**Results:**

Out of 172 patients, 81 reported being abstemious, 45 declared occasional consumption, 11 weekly and 22 daily consumption. There is no data about 13 patients. Among those who reported daily alcohol consumption, 59% had a previous diagnosis of Substance Use Disorder (SUD), 23% a previous diagnosis of Schizophrenia, 13.5% of Bipolar Disorder and finally 4.5% of Depressive Disorder. All the patients with a previous diagnosis of SUD reported consumption of more than 10 SDUs/day, the group with Schizophrenia stated less than 5 SDUs/day, of the group with T. Bipolar between 7-10 SDUs/day and with T. Depressive 5 SDUs/day.

**Conclusions:**

The results obtained are consistent with the literature in relation to the under-diagnosis of alcohol use disorder, taking into account that 40% of patients in the sample with daily alcohol consumption previously had not such a diagnosis and it was not recorded in their medical history. For this reason, and for the sake of being able to treat them, it is essential to question all patients about alcohol consumption, whatever the reason for their admission.

**Disclosure of Interest:**

None Declared

